# Geriatric nutritional risk index as a predictor of outcomes in elderly patients undergoing percutaneous coronary intervention: An updated systematic review and meta-analysis

**DOI:** 10.12669/pjms.41.8.12092

**Published:** 2025-08

**Authors:** Fei Fang, Xiaowang Li

**Affiliations:** 1 Fei Fang Department of Geriatrics, Huzhou Third Municipal Hospital, The Affiliated Hospital of Huzhou University, Huzhou, Zhejiang Province 313000, P.R. China; 2 Xiaowang Li Department of Cardiovascular Interventional Treatment Center, First affiliated Hospital of Huzhou University, Huzhou First People’s Hospital, Huzhou, Zhejiang Province 313000, P.R. China

**Keywords:** Percutaneous coronary intervention, Coronary artery disease, Myocardial infarction, Mortality, Nutritional status

## Abstract

**Objective:**

To assess the association between Geriatric Nutritional Risk Index (GNRI) and outcomes in elderly patients undergoing percutaneous coronary intervention (PCI).

**Methods:**

A systematic search of PubMed, Web of Science, Scopus, and Embase databases was done to identify observational studies that reported adjusted effect sizes for the outcomes of interest, such as all-cause mortality (ACM), major adverse cardiovascular events (MACEs) and contrast-induced acute kidney injury (CI-AKI). Databases were searched from inception of the databases and until April 30, 2024. Subgroup analysis was conducted based on underlying cardiac condition, follow up duration, sample size and quality score. Analysis using random effects model was conducted in STATA version 15.0. Publication bias was assessed using Egger’s test and funnel plot.

**Results:**

A total of 24 studies with 35002 participants were included. Low GNRI scores were associated with the increased risk of ACM (HR 2.50, 95% CI: 2.08, 3.00; n=13, I^2^=39.8%) and MACE (HR 2.18, 95% CI: 1.58, 3.01; n=11, I^2^=95.0%) compared to high GNRI scores. The risk of CI-AKI was higher in patients with low GNRI score (HR 1.87, 95% CI: 1.41, 2.48; n=2, I^2^=0.0%). Significant association of low GNRI scores and increased risk of ACM and MACEs was consistent in all subgroup analyses. Evidence of publication bias was found for ACM and MACEs outcomes.

**Conclusion:**

This study demonstrated a consistent association between low GNRI scores and increased risks of ACM, MACEs and CI-AKI in older patients undergoing PCI. Evidence of publication bias warrants cautious interpretation. Our findings underscore the importance of nutritional assessment and intervention to improve outcomes in at-risk populations.

***PROSPERO Registration No.:*** CRD42024537137).

## INTRODUCTION

With the gradual aging of world population, proportion of people aged 65 years and older has increased globally from 6.1% to 8.8% between 1990 and 2017.^1^ This demographic shift has drawn attention to the prevalence of malnutrition among older individuals who are particularly vulnerable to morbidities.^2^ Recent studies have shown that malnutrition may exacerbate illness, prolong hospital stays, and substantially increase morbidity and mortality rates of patients.^3^,^4^ Notably, malnutrition has been identified as a significant contributor to mortality in patients with heart diseases.^5^,^6^

The Geriatric Nutritional Risk Index (GNRI) has emerged as a promising tool for assessing nutritional status,^7^ and a useful prognostic indicator in patients with various malignancies and cardiac diseases.^8^-^11^ GNRI is a composite measure, incorporating serum albumin levels and body mass index (BMI), and therefore, provides valuable insights into the nutritional status and overall health status of patients.^7^ The tool has been used as a predictor of adverse outcomes in a number of cardiac diseases like congenital heart disease, heart failure, and acute coronary syndrome.^5^,^7^,^11^

Percutaneous coronary intervention (PCI) is considered a cornerstone treatment for coronary artery disease.^12^ However, despite advancements in procedural techniques and adjunctive therapies, PCI is still associated with numerous adverse outcomes, particularly in elderly patients.^13^,^14^ Geriatric population of patients undergoing PCI often presents with multiple comorbidities and age-related physiological changes, which may influence procedural success and post-procedural outcomes.^14^,^15^ In this context, identifying reliable predictors of outcomes following PCI is essential for optimizing patient care and informing clinical decision-making.

Given the established association between malnutrition and adverse outcomes in cardiovascular diseases, there is growing interest in exploring the prognostic value of various malnutrition indices like Controlling Nutritional Status (CONUT), Nutritional Risk Screening 2002, Mini-Nutritional Assessment, and Prognostic Nutritional Index (PNI), and GNRI in patients undergoing PCI.^16^,^17^ A previous review by Fan et al that included eight studies with nearly 9000 patients, showed that low GNRI score was associated with a higher risk of all-cause mortality and adverse cardiovascular events in patients with coronary artery disease.^16^ Another review by He et al.^17^ that included 30 cohort studies in patients with coronary artery disease, found that malnutrition was associated with an increased risk of all-cause mortality and major adverse cardiovascular events. While, the review did not focus specifically on GNRI and PCI, it showed that GNRI was a better predictor of adverse outcomes than other indices.^17^

This study aimed to consolidate and update existing evidence on the association of GNRI with outcomes after PCI in elderly patients, irrespective of the underlying cardiac condition necessitating PCI. The primary outcomes for this analysis included all-cause mortality, major adverse cardiovascular events (MACE), and contrast induced acute kidney injury (CI-AKI).

## METHODS

### Search strategy and selection of studies:

Study protocol was registered at PROSPERO (registration number CRD42024537137). PubMed, Web of Science, Scopus, and Embase databases were searched by two reviewers using specific keywords: (Geriatric Nutritional Risk Index [Mesh] OR GNRI OR Nutrition Assessment [Mesh]) OR Nutritional Risk Index OR Malnutrition) AND (Percutaneous Coronary Intervention [Mesh] OR PCI) AND (adverse outcomes OR complications OR mortality OR MACE OR CIN OR nephropathy OR adverse cardiac events). The search strategy was customized as per the needs of each of the databases. Search scope was from inception until April 30, 2024. The PRISMA guidelines were followed.^18^ We also checked the reference list of included studies for any missed article.

Identification of eligible studies was done independently by two study authors (FF, XL). The initial pool of studies obtained by the literature search of the databases was reviewed and deduplicated. Titles and abstracts of the remaining studies were examined to identify relevant publications. Full text of these studies was then reviewed to identify the final pool of eligible studies that were included in the review. Disagreements were resolved after discussion.

### Inclusion & Exclusion Criteria:

We included studies which fulfilled the PECOS criteria as follows:

1. Population: were conducted on a study population of older patients (aged 65 years or above) undergoing PCI, regardless of their underlying cardiac condition.
2. Exposure & Comparison: which assessed the relationship between pre-PCI GNRI and patient outcomes.
3. Outcomes: Reported on at least one of the following outcomes: all-cause mortality, MACE, and contrast-induced AKI. For studies examining MACE, explicit definitions detailing clinical conditions included in this composite outcome were necessary. Reported outcomes should have been assessed during follow-up after PCI, with a minimum follow-up period of three months.
4. Study design: were cohort, cross-sectional, and case-control studies, and published in peer-reviewed journals

We excluded studies where GNRI measurements were not obtained prior to PCI or were assessed only after the procedure or during the follow-up period. Additionally, review articles, editorials, conference abstracts, letters to the editor, and case reports were excluded. Duplicate publications or studies with overlapping datasets were also excluded.

### Data extraction:

Extraction of relevant data from the final set of studies was independently carried out by the two authors using a structured extraction form, developed by the authors through mutual discussion. All discrepancies and disagreements were resolved through discussions.

### Quality assessment:

The Newcastle-Ottawa Scale (NOS) was used for assessing quality of the studies.^19^ The NOS is divided into three domains: cohort representativeness, comparability, and outcome measurement. Points are awarded depending on the questions listed in the NOS. The maximum score which can be awarded is nine.

### Statistical analysis:

Pooled effect sizes were reported as Odds ratio (OR) or hazard ratios (HR), along with 95% confidence intervals (CI). Random-effects model was used to account for differences in participant characteristics and any methodological variations among the included studies.^20^ The analysis was conducted with both GNRI as a categorical exposure (high and low) and a continuous exposure (scores not categorized). Publication bias was assessed through Egger’s test.^21^ Subgroup analysis was conducted based on sample size (≤500 and >500), underlying cardiac condition, duration of follow up (≤ two years and > two years) and NOS score (≥eight and ≤seven). Heterogeneity was assessed using I^2^ statistics with values >50% indicating high heterogeneity. A p-value below 0.05 was considered statistically significant. All analysis was conducted using STATA version 15.0.

## RESULTS

**Fig.1:**
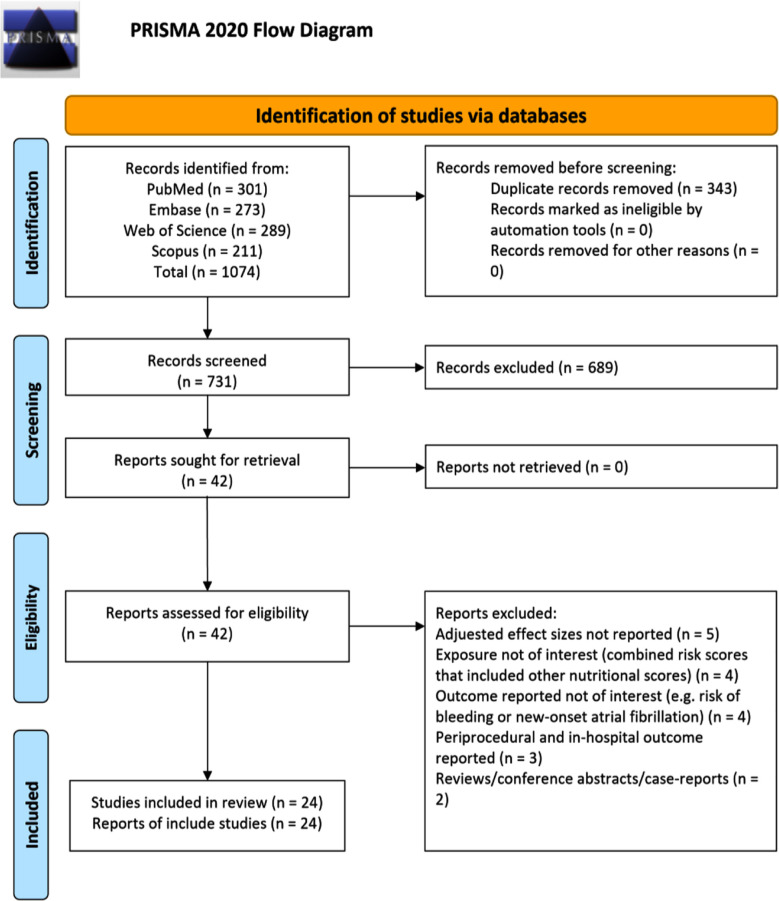
PRISMA flowchart to show process of study selection.

Systematic search across the specified databases led to retrieval of 1074 studies. Of them, 343 duplicates were removed, and 731 unique studies were screened based on titles and abstracts. Fig.1 Additional 689 studies were then excluded, and full texts of the remaining 42 studies were carefully reviewed. Of them, 18 studies were excluded. Finally, 24 studies were considered eligible for inclusion in the meta-analysis.^22^-^45^

### Characteristics of the included studies:

Overall, the included studies contributed to a sample of 35002 patients. Out of the maximum attainable score of Nine on NOS, all studies obtained a score of ≥7 Supplementary Table-I. Almost all studies were cohort in design (n=23) and were conducted in Asian settings. Mean age of the patients ranged from 65 to 80 years, with higher proportion of male participants (50 to 85%) Table-I. The follow up period for assessment and reporting of the outcomes ranged from 12 months to 7.5 years. Among studies that reported on the duration of follow up, 14 had a follow up duration of more than two years. Primary cardiac condition necessitating PCI was acute coronary syndrome (ACS) in six studies, coronary artery disease (CAD) in six studies and acute myocardial infarction (AMI) in nine studies. The cut-off used by the studies to define high and low GNRI score varied considerably, with no standardized criteria. Similarly, operational definition of MACE varied among the studies.

**Supplementary Table-I:** Summary of the included studies.

Author, study design and location	Age, sex and primary cardiac condition	Cut-off of GNRI for low and high risk of malnutrition	Variables adjusted in the analytic model	Size of the sample studied	Duration of follow-up	Important outcomes assessed	Newcastle Ottawa Scale score
Zhu (2024)^22^ Cohort; China	Mean age 65 years Male (75%) Acute coronary syndrome	High risk (<111) Low risk (≥111)	Age, GRACE score, Systemic Immune Inflammatory Index score	827	1 year	MACE* *Included recurrent angina, severe arrhythmia, rehospitalization for cardiovascular reasons, acute myocardial infarction, heart failure, and death from coronary heart disease	S-4 C-2 O-2
Sun (2023)^23^ Cohort; China	Mean age 60 years Male (83%) Ischemic heart failure	High risk (<99.9) Low risk (≥107.7)	Age, gender, systolic blood pressure, heart rate, NYHA class, hypertension, atrial fibrillation, hypercholesterolemia, prior MI, prior PCI, haemoglobin, triglyceride, blood nitrogen urea, eGFR	871	Mean 28.7 months	MACE* ACM *Included all-cause mortality, non-fatal myocardial infarction (MI), and any revascularization	S-4 C-2 O-2
Lim (2023)^24^ Cohort; Republic of Korea	Higher mean age in those with low GNRI group (72 vs. 60 years); lower proportion of males in low GNRI group (67% vs. 79%) Acute myocardial infarction (AMI)	High risk (<82) Low risk (≥98)	Age, sex, body mass index, hypertension, dyslipidemia, diabetes, prior MI, prior PCI, prior stroke, atrial fibrillation, diagnosis, eGFR, left ventricular ejection fraction (LVEF)	5312	Median 4.9 years	MACE* ACM *Included cardiovascular death, recurrent myocardial infarction (MI), and ischemic stroke	S-4 C-2 O-2
Chen (2023)^25^ Cohort; China	Mean age 65 years Male (80%) Unstable angina (50%) and NSTEMI (19%)	High risk (<92) Low risk (>98) Also analysed as continuous variable	Age, sex, diabetes, heart failure, hypertension, anemia, eGFR, acute myocardial infarction, multivessel disease, emergency PCI, systolic blood pressure, white blood cells, glucose, low-density lipoprotein cholesterol, and uric acid.	6049	Not provided	CI-AKI	S-4 C-2 O-1
Anzaki (2023)^26^ Cohort; Japan	Mean 69 years Male (71%) Coronary artery disease	High risk (<92) No risk (≥92)	Age, sex, haemodialysis, dyslipidaemia, statin, hs-CRP, eGFR	500	Median of 537 days	ACM MACE* *Included all-cause death, non-fatal myocardial infarction, and ischemic stroke	S-4 C-2 O-2
Nakamura (2022)^27^ Cohort; Japan	Mean 81 years Male (55%) Acute MI	High risk (<92) No risk (≥92)	Age, sex, diabetes, hypertension, natriuretic peptide, Killip class, smoking	130	Mean of 1030 days	ACM	S-4 C-2 O-2
Kanda (2022)^28^ Cohort; Japan	Mean age 68 years Male (69%) Acute myocardial infarction (AMI)	High risk (<92) Low risk (≥92)	Age, LVEF, GRACE risk score	268	Median 698 days	ACM	S-4 C-1 O-2
Li (2022)^29^ Cross sectional; China	Mean age 67 years Male (66%) Not reported	High risk (<90) Low risk (≥104)	Age, gender, diabetes, blood pressure, eGFR, LVEF, haemoglobin, C-reactive protein, the volume of contrast agent consumption, type of contrast agent, pre-procedure medication	4386	Not required as it’s a cross-sectional study	CI-AKI	S-4 C-2 O-1
Chen (2022)^30^ Cohort; China	Mean age 66 years Male (72%) Acute coronary syndrome	GNRI as a continuous variable	Sex, GRACE score, previous coronary artery disease, diabetes, hypertension, triglyceride, serum albumin, total lymphocyte count, weight loss	799	Median 30 months	ACM	S-4 C-2 O-1
Yıldırım (2021)^31^ Cohort; Turkey	Mean age 73 years Male (52%) Non-ST-elevated myocardial infarction (NSTEMI)	GNRI as a continuous variable	Age, sex, body mass index, hypertension, triglycerides, total protein, left ventricular ejection fraction	915	Mean 64.5 months	ACM	S-4 C-2 O-1
Arikawa (2021)^32^ Cohort; Japan	Mean age 70 years Male (68%) Stable coronary artery disease and myocardial damage	High risk (<92) Low risk (≥92)	Age, sex, body mass index, hypertension, diabetes, dyslipidemia, prior MI, prior heart failure, smoking	241	Mean 546 days	MACE* *Included all-cause death, non-fatal myocardial infarction, and ischemic stroke	S-4 C-2 O-2
Kaplan (2021)^33^ Cohort; Turkey	Mean age 72 years Male (73%) NSTEMI	Moderate to High risk (82 to <92) No/low risk (>98)	Multivariate Cox regression analysis performed but variables included for adjustment not mentioned	298	Mean 32.9 months	ACM	S-4 C-2 O-1
Cheng (2021)^34^ Cohort; China	Mean age 65 years Male (80%) CTO	Moderate to High risk (<92) No risk (≥98)	Age, sex, lifestyle factors (smoking, alcohol), hypertension, diabetes mellitus, cholesterol level, LVEF, pro-BNP, creatinine, hsCRP	472	Median of 33 months	ACM MACE* *Included all-cause death, nonfatal acute myocardial infarction, revascularization and stroke.	S-4 C-2 O-2
Kobayashi (2021)^35^ Cohort; Japan	Mean age 65 years Male (75%) Acute coronary syndrome	High risk (≤98) No risk (>98)	Age, sex, diabetes, haemoglobin, Killip class, three vessel disease, haemodialysis	1461	Median of 1219 days	ACM	S-4 C-2 O-1
Kim (2021)^36^ Cohort; Republic of Korea	Mean age 66 years Male (73%) Acute MI	High risk (<103.8) No risk (>112.3) Also analysed as continuous variable	Age, sex, current smoker, creatinine, hypertension, diabetes, BMI, ejection fraction, multivessel disease	1147	Follow up 12 months	ACM MACE* *Included cardiac death, re-AMI, revascularization, CHF, and CVA.	S-4 C-2 O-2
Ma (2021)^37^ Cohort; China	Mean 60 years Male (77%) Acute coronary syndrome	High risk (<97.5) No risk (≥100) Also analysed as a continuous variable	Clinical variables, coronary revascularization, optimal medical treatment, lymphocyte count, neutrophil count, monocyte count, hs-CRP, GRACE score, sex, BMI, current smoking, family history of CAD, hypertension, dyslipidaemia, diabetes, past MI, past PCI	1510	Median of 927 days	MACE* *Included all-cause death, non-fatal stroke, non-fatal MI, or unplanned repeat revascularization	S-4 C-2 O-1
Zhao (2020)^38^ Cohort; China	Mean age 60 years Male (72%) Acute coronary syndrome	High risk (<103.6) No risk (≥103.6) Also analysed as a continuous variable	Age, sex, BMI, hypertension, diabetes, LVEF, c-reactive protein, eGFR	2299	Follow up of 3 years	MACE* ACM *Included all-cause death, non-fatal myocardial infarction (MI), and any revascularization	S-4 C-2 O-1
Jia (2020)^39^ Cohort; China	Mean age 64 years Male (77%) STEMI	GNRI analysed as a continuous variable	Age, LVEF, c-reactive protein, haemoglobin, Killip class, blood-urea nitrogen, blood glucose	786	Median 12.4 months	MACE* *Included cardiovascular related mortality	S-4 C-2 O-1
Kucukosmanoglu (2020)^40^ Cohort; Turkey	Mean age 61 years Male (67%) NSTEMI	GNRI analysed as a continuous variable	Age, diabetes, albumin, LVEF, haemoglobin, triglyceride, high density cholesterol (HDL-C)	1116	Not provided	CI-AKI	S-4 C-2 O-1
Katayama (2020)^41^ Cohort; Japan	Mean age 74 years Male (63%) Rotational atherectomy for calcified lesions	High risk (<98) No risk (≥98)	Age, sex, hemodialysis, total lymphocytes, serum albumin, BMI, and a history of PCI.	206	Follow up of 12 months	MACE* *Included all-cause death, target lesion revascularization (TLR), target vessel revascularization (TVR), and myocardial infarction.	S-4 C-2 O-1
Ando (2020)^42^ Cohort; Japan	Mean age and sex distribution not provided Acute MI	High risk (<92) No risk (≥98)	Cox proportional hazard analysis was done with adjustment for potential confounding factors: the variables adjusted for have not be mentioned	552	Mean of 1424 days	ACM	S-4 C-2 O-1
Raposeiras (2020)^43^ Cohort; Spain	Mean age 66 years Male (75%) Acute coronary syndrome	High risk (<83.5) No risk (≥100) Also analysed as continuous variable	Age, sex, body mass index, hypertension, dyslipidaemia, diabetes, prior myocardial infarction, prior heart failure, peripheral artery disease, GRACE score	2228	Median 3.6 years	ACM MACE* *Included cardiovascular death, reinfarction, or ischemic stroke	S-4 C-2 O-2
Wada (2017)^44^ Cohort; Japan	Higher mean age in those with low GNRI group (69 vs. 62 years); lower proportion of males in low GNRI group (76% vs. 88%) Coronary artery disease (CAD)	High risk (<98) No/low risk (>104) Also analysed as continuous score	Acute coronary syndrome, age, chronic kidney disease, diabetes mellitus, high-sensitivity C-reactive protein, left ventricular ejection fraction, multivessel coronary disease, and use of statins	1942	Median 7.4 years	ACM	S-4 C-2 O-2
Kunimura (2017)^45^ Cohort; Japan	Higher mean age in those with low GNRI group (75 vs. 69 years); lower proportion of males in low GNRI group (64% vs. 71%) Coronary artery disease (CAD)	Moderate to High risk (<92) No risk (>98)	Age, sex, current smoker, diabetes mellitus, hypertension, dyslipidemia, eGFR, statins, brain natriuretic peptide; eGFR	687	Median of 1568 days	MACE* *Included cardiac death and/or non-fatal myocardial infarction .	S-4 C-2 O-1

MACE: Major adverse cardiovascular events; ACM: All-cause mortality; CI-AKI: Contrast induced acute kidney injury; NYHA: New York Heart Association; MI: myocardial infarction; PCI: percutaneous intervention; eGFR: estimated glomerular filtration rate; CTO: chronic total occlusion; NSTEMI: Non-ST-elevation myocardial infarction; STEMI: ST-elevation myocardial infarction; AMI: acute myocardial infarction; CHF: congestive heart failure; CVA: cerebrovascular attack; S-Selection of cohort; C-Comparability of groups; O-Outcome assessment

**Table-I:** Subgroup analysis.

	All-cause mortality	MACE
	Pooled HR (95% CI) (Number of studies; I^2^)	
***Cardiac disease***		
ACS	2.75 (2.14, 3.53) (N=3; 0.0%) *	2.24 (1.67, 3.00) (N=4; 72.7%) *
Acute MI	2.83 (1.91, 4.19) (N=6; 60.4%) *	1.67 (1.42, 1.96) (N=1; ---) *
CAD	2.35 (1.62, 3.41) (N=3; 25.9%) *	2.26 (1.17, 4.33) (N=5; 89.4%) *
***Sample size***		
>500	2.22 (1.88, 2.63) (N=8; 26.9%) *	2.36 (1.83, 3.04) (N=7; 80.6%) *
≤500	3.41 (2.43, 4.79) (N=5; 0.0%) *	1.72 (1.02, 2.89) (N=4; 80.7%) *
***Follow up***		
≤2 years	3.64 (2.23, 5.94) (N=3; 0.0%) *	2.09 (1.00, 4.36) (N=4; 94.3%) *
>2 years	2.37 (1.96, 2.85) (N=10; 40.4%) *	2.17 (1.71, 2.75) (N=7; 75.2) *
***NOS score***		
≥8	2.28 (1.91, 2.73) (N=8; 34.2%) *	2.27 (1.81, 2.84) (N=7; 66.0%) *
≤7	3.28 (2.21, 4.87) (N=5; 18.3%) *	2.04 (1.14, 3.63) (N=4; 94.6%) *

* indicates statistical significance at p<0.05; MACE: Major adverse cardiovascular events; ACS: acute coronary syndrome; CAD: coronary artery disease; MI: myocardial infarction; NOS: Newcastle Ottawa Scale Score.

### All-cause mortality:

**Fig.2:**
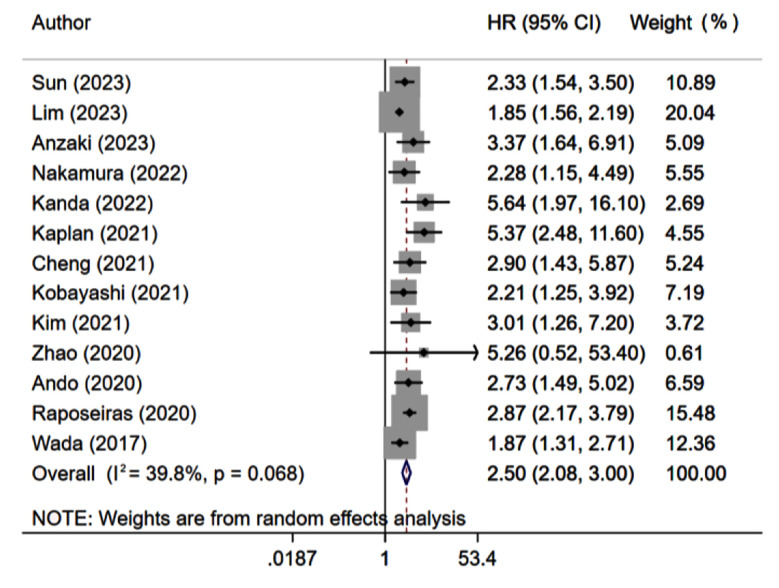
Risk of all-cause mortality among those with low GNRI score, compared to those with high GNRI score.

Compared to patients with high GNRI scores, patients with low GNRI scores were at increased risk of all-cause mortality (HR 2.50, 95% CI: 2.08, 3.00; n=13, I^2^=39.8%) (Fig.2). This significant association of low GNRI scores with increased risk of mortality was found consistently in subgroup analyses based on the sample size, underlying cardiac condition, duration of follow up and quality score (Table-I). There was evidence of publication bias on Egger’s test (p=0.006). Analysis with GNRI scores as a continuous variable showed that each unit increase in score was associated with a statistically significant decrease in the risk of all-cause mortality (HR 0.94, 95% CI: 0.92, 0.97; n=6, I^2^=85.3%).

### Major adverse cardiovascular events (MACEs):

**Fig.3:**
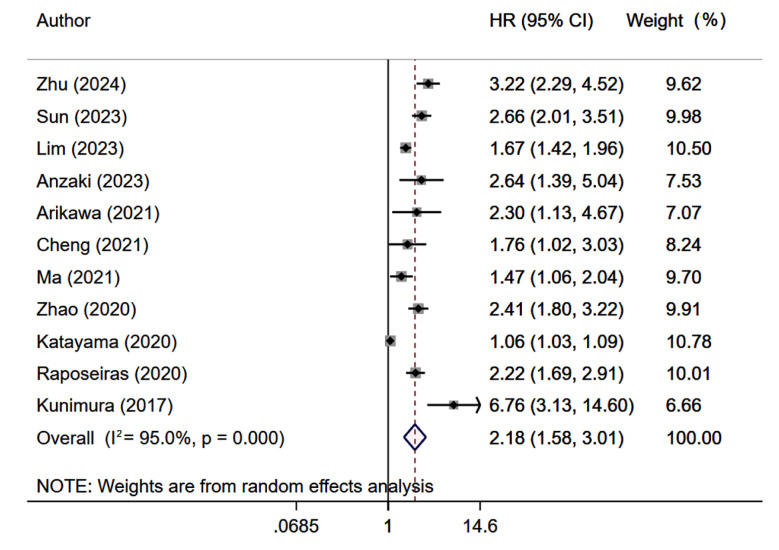
Risk of major adverse cardiovascular events (MACEs) among those with low GNRI score, compared to those with high GNRI score.

Patients with low GNRI scores were at higher risk of developing MACE (HR 2.18, 95% CI: 1.58, 3.01; n=11, I^2^=95.0%), compared to patients with high scores. Fig.3 Subgroup analyses across different categories based on sample size, underlying cardiac condition, duration of follow up and quality score showed similar findings Table-I. The Egger’s test (p=0.001) suggested presence of publication bias. Each unit increase in GNRI score was associated with decreased risk of developing MACE (HR 0.93, 95% CI: 0.89, 0.98; n=5, I^2^=94.9%).

### Contrast-induced acute kidney injury (CI-AKI):

**Fig.4:**
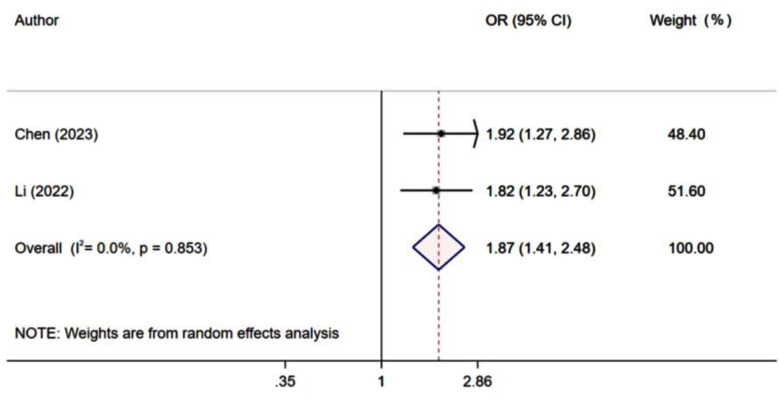
Risk of contrast induced acute kidney injury (CI-AKI) among those with low GNRI score, compared to those with high GNRI score.

Risk of CI-AKI was significantly higher in patients with low GNRI score (HR 1.87, 95% CI: 1.41, 2.48; n=2, I^2^=0.0%) Fig.4. Furthermore, each unit increase in GNRI score correlated with a significant reduction in the risk of CI-AKI (HR 0.76, 95% CI: 0.72, 0.80; n=2, I^2^=0.0%). Assessment for publication bias could not be done due to limited number of studies reporting on this outcome.

## DISCUSSION

The present study is the first meta-analysis of literature focussing on the prognostic ability of GNRI in patients undergoing PCI. Our findings suggest an association between GNRI scores and all-cause mortality, MACE, and CI-AKI in patients undergoing PCI. The study indicates that patients with low GNRI scores are at increased risk of experiencing these adverse events compared to patients with high GNRI scores. This association remained consistent across various subgroup analyses, implying robustness in the observed relationship.

Several instruments have been described in the literature to identify patients as malnourished prior to any intervention or treatment, as quantifying nutritional status has been a challenge. On the one hand, there are straightforward indices such as albumin and body mass index, while on the other hand, there are intricate tools such as the Patient-Generated Subjective Global Assessment and the Subjective Global Assessment tools, which are both difficult to implement in routine practice and require a significant amount of time.^5^,^6^,^17^ Consequently, the PNI, GNRI, Mini-Nutrition Assessment, Malnutrition Universal Screening Tool, and CONUT have been created to facilitate the rapid evaluation of pre-treatment nutritional status by combining readily accessible indices.^6^,^17^ To date, no single optimal nutrition assessment tool has been identified, despite the abundance of nutritional assessment tools available to the treating physician. The GNRI, which was initially introduced in 2005, is a nutritional marker that is being widely used because of its simplicity, easy availability, and good prognostic value.^7^ Similar to our review, prior meta-analysis studies have also identified GNRI as a predictor of various cardiovascular diseases. Low GNRI values are associated with a two-fold increase in the risk of mortality and 2.8 times increase risk of MACE in patients with acute coronary syndrome.^16^ Dong et al.^6^ in a pooled analysis of nine studies have demonstrated that low GNRI values are associated with 59% increase in the risk of mortality in patients with heart failure. Another meta-analysis shows that GNRI is associated with a 3.6 times increase in the risk of short-term mortality and 2.3 times increase in the risk of long-term mortality.^9^ The present review extends the validity of GNRI to cardiac patients undergoing PCI thereby providing robust evidence to clinicians.

A pertinent point is the heterogeneity of the included studies in the meta-analysis which included patients with different diagnoses, follow-ups and sample sizes. Meta-analyses of all outcomes had high heterogeneity which could be partly due to such variations between studies as well as unmeasured differences between the patients like severity of disease, comorbidities, PCI techniques, access routes, type of stents, etc. Lack of detailed and segregated data from the included studies precluded a comprehensive subgroup analysis. However, we could still segregate studies based on diagnosis, sample size, follow-up, and NOS score and noted that low GNRI was still associated with significantly increased risk of adverse events across all subgroups. Importantly, the strength of the association was also high with the pooled HR >1.5 across all subgroups.

The association between low GNRI scores and increased risk of adverse outcomes can be explained through several interconnected mechanisms. Firstly, malnutrition, as indicated by low GNRI scores, often leads to compromised immune function, leaving patients more susceptible to infections and inflammation, both of which contribute significantly to mortality and adverse health outcomes.^46^ Secondly, malnutrition is a recognized risk factor for cardiovascular disease, potentially contributing to conditions like hypertension and dyslipidemia that serve as precursors to MACE.^47^,^48^ Furthermore, malnutrition contributes to chronic inflammation and oxidative stress, which damage vascular endothelium, promote atherosclerosis, and impair renal function, thereby increasing the likelihood of adverse cardiovascular events and kidney injury.^49^-^51^ Malnutrition may also exacerbate medication-related adverse effects, including nephrotoxicity, particularly relevant in individuals with compromised renal function.^52^ Studies show that malnutrition often coexists with chronic diseases such as heart failure and diabetes, further increasing the risks of adverse outcomes.^53^,^54^ Therefore, addressing malnutrition and optimizing nutritional status in vulnerable populations emerges as a crucial strategy to mitigate these interconnected risks and improve overall health outcomes.

### Limitations:

We have reported potential presence of publication bias for most of the outcomes. Publication bias could arise from various factors, such as selective reporting of studies with positive findings or preferential publication of studies with larger effect sizes, and may indicate a potential overestimation of effect sizes. While this bias may influence the overall interpretation of the results, the consistency of the findings across subgroup analyses lends credibility to the observed associations. Further, many included studies were observational, hindering the establishment of causal relationships. Variability in sample characteristics, cut-offs used to define low and high GNRI and definition of outcome measures across studies, particularly for MACE could have introduced heterogeneity and limit generalizability of our results. Further, most of included studies were from Asian setting and therefore, they could not be applicable to other geographical settings. While we used adjusted effects sizes for our analysis, some unaccounted confounding factors might have influenced observed associations. Addressing these limitations through rigorous prospective studies with standardized methodologies and adequate control for confounders is crucial for enhancing the evidence base.

### Study implications:

Our study suggests that routine screening and assessment of nutritional status using tools like the GNRI should become standard practice, particularly among older adults and individuals with cardiovascular or renal conditions, enabling early identification of malnutrition. GNRI can be easily used by clinicians to identify high-risk groups and formulate individualized treatment strategies.

### Implications for future research:

Mechanistic studies are needed to elucidate the underlying biological pathways linking malnutrition, as indicated by low GNRI scores, to outcomes such as mortality, MACEs, and AKI. Longitudinal investigations can provide insight into the long-term impact of nutritional interventions on health outcomes in at-risk populations. Interventional trials are essential for evaluating the efficacy of specific interventions, including dietary modifications and nutritional supplementation, in improving outcomes.

## CONCLUSION

Our results suggest that low pre-procedural GNRI scores are associated with increased risks of all-cause mortality, MACEs and CI-AKI in older patients undergoing PCI. Evidence of publication bias warrants cautious interpretation. Further high-quality studies shall provide robust evidence.

### Authors’ contributions:

**FF and XL:** Study design. Literature search and manuscript writing.

**FF and XL:** Data collection, data analysis and interpretation. Critical Review

**FF:** Revision and validation, critical analysis and is responsible for the integrity of the study.

All authors have read and approved the final manuscript.
